# Some Like It Hot: Dynamic Control of Cav2.2 Channels By Chili Peppers

**DOI:** 10.1093/function/zqac066

**Published:** 2022-12-24

**Authors:** Paz Duran, Rajesh Khanna

**Affiliations:** Department of Molecular Pathobiology, College of Dentistry New York University, 433 First Avenue, 8th floor, New York, NY 10010, USA; NYU Pain Research Center, New York University, 433 First Avenue, 8th floor, New York, NY 10010, USA; Department of Molecular Pathobiology, College of Dentistry New York University, 433 First Avenue, 8th floor, New York, NY 10010, USA; NYU Pain Research Center, New York University, 433 First Avenue, 8th floor, New York, NY 10010, USA

## A Perspective on “Capsaicin-induced Endocytosis of Endogenous Presynaptic CaV2.2 in Dorsal Root Ganglion (DRG)-spinal Cord Co-Cultures Inhibits Presynaptic Function”

Spicy meals causes the production of happy endorphins together with the triggering of heat and pain, similar to a runner’s high. The active ingredient in hot chili peppers that causes their distinctive burning sensation is called capsaicin (8-methyl-N-vanillyl-6-nonenamide). This bioactive substance binds to the primary afferent neurons’ transient receptor potential vanilloid 1 (TRPV1) cation channels, which when activated, cause a sensation of heat. Capsaicin has been utilized as a tool to study the regulation of pain since TRPV1 channels have been reported to be crucial for heat nociception.^1^ Despite reports that capsaicin binding to TRPV1 channels causes pain, it has been demonstrated that prolonged exposures to capsaicin can *desensitize* dorsal root ganglion (DRG) neurons, thus reducing afferent drive and reducing synaptic transmission in the dorsal horn.^2^

Several studies have established that voltage-gated calcium channels (VGCCs) are key modulators of nociceptive and nociplastic pain.^3^ VGCCs are transmembrane proteins composed of a principal pore-forming α subunit that mediates Ca^2+^ entry into the cell in response to membrane potential changes. Based on their biophysical characteristics, VGCCs are classified into low voltage activated (LVA) and high voltage activated (HVA) families. HVA channels are typically expressed with auxiliary subunits β and α2δ that regulate the trafficking and function of these channels. The N-type calcium channel, also known as CaV2.2, is a member of the HVA family that is expressed at high levels in sensory neurons where they are key mediators of neurotransmitter release and the transmission of sensory information from the periphery to central sites.^4^ Given that CaV2.2 channels are the main presynaptic VGCCs and have a critical role in regulating nociceptive transmission, it is reasonable to predict a regulation mediated by capsaicin and TRPV1. However, little is known about the underlying mechanisms of the functional interaction between these channels and their presynaptic function. This gap in knowledge was explored in a very ingenious way by Krishma Ramgoolam and Annette Dolphin in a new study reported in this issue of FUNCTION.^5^The authors build on their long-standing expertise of N-type calcium channels (CaV2.2) to investigate their functional presynaptic expression and explore their interaction with TRPV1 channels in primary nociceptors. Here, the Dolphin group used their previously described CaV2.2_HA knock-in mouse line, which expresses CaV2.2 with a hemagglutinin (HA) exofacial epitope tag to easily localize endogenous CaV2.2 channels.^5^ Using co-cultures of DRG neurons isolated from CaV2.2_HA knock-in mice with spinal cord neurons from wild-type (WT) mice and approaches, including immunofluorescence staining and calcium imaging, this study investigated the neuronal maturation, synapse formation, distribution, and presynaptic function of the tagged N-type calcium channels.

First, CaV2.2 localization during neuronal maturation and synapse formation was explored using immunofluorescence staining. CaV2.2_HA expression over time showed a decrease in cell surface expression at the cell body of DRG neurons that was accompanied by a commensurate significant increase at presynaptic terminals. The authors then extended the characterization of the development of CaV2.2_HA expression at presynaptic boutons using super-resolution microscopy and found that, in mature cultures, the channels were closely juxtaposed to synaptic markers Homer, RIM 1/2 and vGlut2, suggesting the formation of mature synapses. Next, using live-cell calcium imaging experiments, the authors showed that these synaptic boutons were functional. Recordings of Ca^2+^ transients in response to 1 action potential stimulation demonstrated that N-type calcium channels are responsible for a large proportion of the total Ca^2+^ transient amplitude.

The examination of CaV2.2_HA expression pattern in these co-cultures indicated that they are highly expressed in TRPV1-positive medium DRG neurons. These data are consistent with reports of a cross-talk between these channels in primary afferent sensory neurons where capsaicin mediated TRPV1 activation has been reported to cause a reduction in N-type Ca^2+^ currents.^6–8^ Hence, to study the changes in presynaptic localization and function of CaV2.2 channels in response to the activation of TRPV1 channels in primary nociceptors, the authors evaluated the response to capsaicin treatment. The exciting results showed that a 2 min incubation with capsaicin caused a pronounced reduction in cell surface expression of CaV2.2_HA channels in small and medium diameter DRG neurons. Furthermore, capsaicin treatment also decreased CaV2.2 channel’s colocalization with the presynaptic marker RIM 1/2. Additionally, pre-treatment with capsaicin reduced the percentage of N-type contribution to Ca^2+^ transients with a slow timescale. However, only a small decrease was observed in peak Ca^2+^ transients after capsaicin treatment when compared to the control condition. The investigators propose that it is likely that there could be some compensation from other VGCCs, such as CaV2.1 (P/Q-type) or Cav2.3 (R-type) channels.

Trafficking and endocytosis are well studied molecular mechanisms that direct functional expression of VGCCs. In this study, the effect of capsaicin pre-treatment on CaV2.2_HA expression was partially inhibited by reduced temperature (17°C)—which has been known to affect trafficking and endocytosis of many proteins—implicating endocytosis in CaV2.2 regulation. Based on this observation and given that it has been reported previously by the Dolphin group that CaV2.2 channels and their auxiliary subunit (α2δ-1) membrane localization are regulated by Rab11a-dependent endosomal recycling,^9^ the authors hypothesized that this endosomal pathway was involved in the capsaicin-induced CaV2.2 regulation. To address this, the authors used both immunolabeling and calcium imaging experiments using DRG neurons transfected with a dominant-negative Rab112a (S25N). Their results unequivocally showed that CaV2.2 endocytosis in response to capsaicin treatment was prevented in Rab112a S25N transfected DRG neurons.

**Figure 1.**
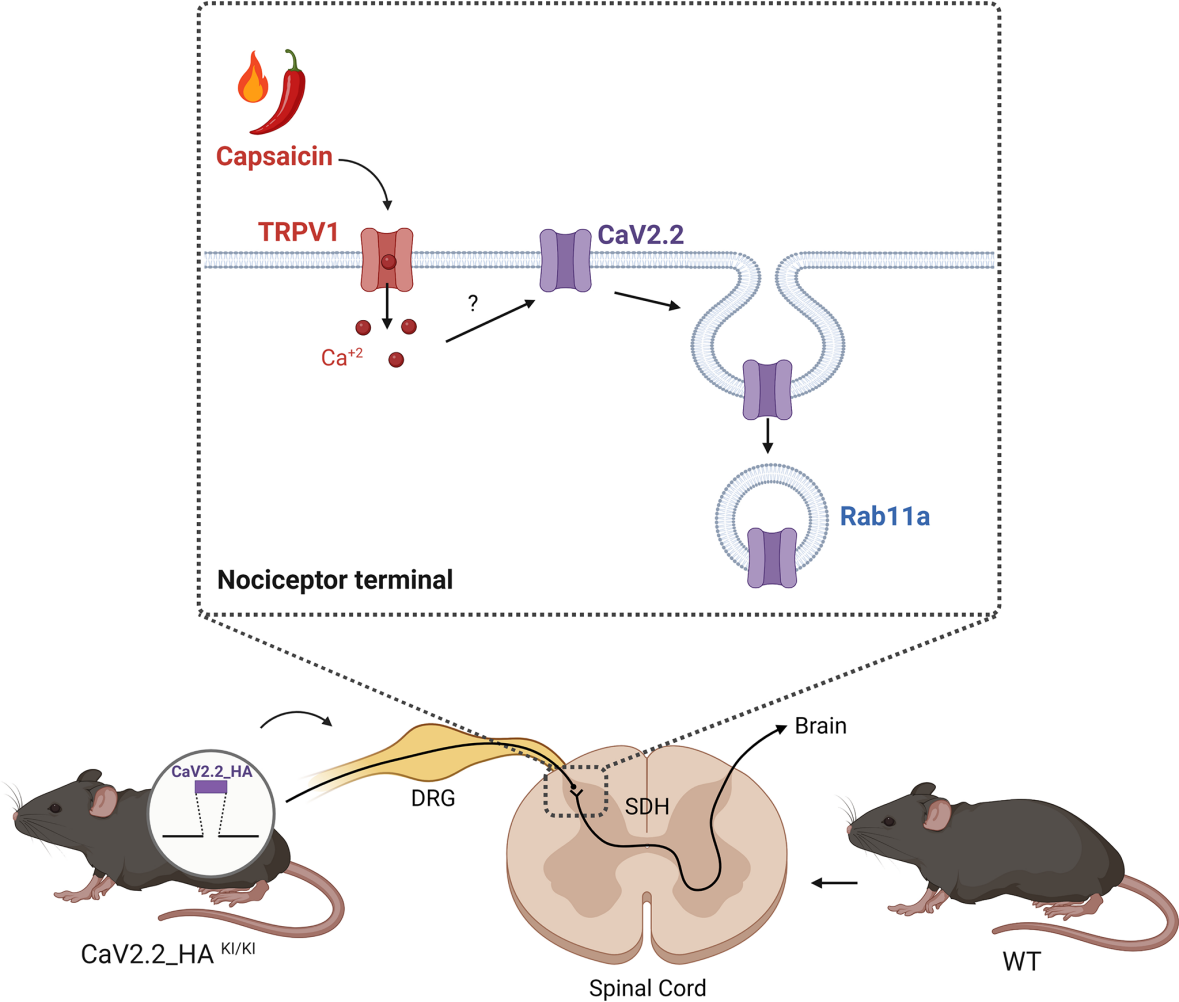
Dynamic regulation of presynaptic CaV2.2 by capsaicin. Schematic diagram representing CaV2.2 regulation at presynaptic sites in response to capsaicin-induced TRPV1 channel activation. Co-cultures of dorsal root ganglion (DRG) neurons isolated from CaV2.2_HA knock-in mice (CaV2.2_HA^KI/KI^) with wild-type (WT) spinal cord neurons were used to study CaV2.2 dynamic regulation by capsaicin. Brief application of capsaicin, a TRPV1 agonist, promotes CaV2.2 downregulation involving Rab11a-dependent endosomal trafficking. Figure created with BioRender.com.

In summary, this is the first study examining the membrane expression and function of CaV2.2 channels at presynaptic sites and the effect of capsaicin-induced TRPV1 channel activation on N-type calcium channels’ functional expression (Figure 1). The results presented provide important new insights into the dynamic regulation of the localization and recycling of presynaptic CaV2.2 channels in response to a brief exposure to capsaicin. Some of the implications of this study are the understanding of the function of these channels in the nociceptive pathway for the further development of therapeutic tools for treating chronic pain.
